# Extracellular DNA-induced antimicrobial peptide resistance mechanisms in *Pseudomonas aeruginosa*

**DOI:** 10.3389/fmicb.2013.00021

**Published:** 2013-02-14

**Authors:** Shawn Lewenza

**Affiliations:** ^1^Snyder Institute for Chronic Diseases, University of CalgaryCalgary, AB, Canada; ^2^Department of Microbiology, Immunology and Infectious Diseases, University of CalgaryCalgary, AB, Canada

**Keywords:** antibiotic resistance, antimicrobial peptides, biofilm, PhoPQ, PmrAB, *Pseudomonas aeruginosa*, immune evasion, extracellular DNA

## Abstract

Extracellular DNA (eDNA) is in the environment, bodily fluids, in the matrix of biofilms, and accumulates at infection sites. eDNA can function as a nutrient source, a universal biofilm matrix component, and an innate immune effector in eDNA traps. In biofilms, eDNA is required for attachment, aggregation, and stabilization of microcolonies. We have recently shown that eDNA can sequester divalent metal cations, which has interesting implications on antibiotic resistance. eDNA binds metal cations and thus activates the Mg^2+^-responsive PhoPQ and PmrAB two-component systems. In *Pseudomonas aeruginosa* and many other Gram-negative bacteria, the PhoPQ/PmrAB systems control various genes required for virulence and resisting killing by antimicrobial peptides (APs), including the *pmr* genes (*PA3552*–*PA3559*) that are responsible for the addition of aminoarabinose to lipid A. The *PA4773*–*PA4775* genes are a second DNA-induced cluster and are required for the production of spermidine on the outer surface, which protects the outer membrane from AP treatment. Both modifications mask the negative surface charges and limit membrane damage by APs. DNA-enriched biofilms or planktonic cultures have increased antibiotic resistance phenotypes to APs and aminoglycosides. These dual antibiotic resistance and immune evasion strategies may be expressed in DNA-rich environments and contribute to long-term survival.

## SOURCE AND FUNCTIONS OF EXTRACELLULAR DNA

Extracellular DNA (eDNA) is released from dead plant or microorganisms and accumulates in soil, aquatic, and sediment environments (Dell’Anno and Danovaro, 2005; Vlassov et al., 2007; Pietramellar et al., 2009). Bacteria actively release or secrete DNA, or it is released during bacterial lysis and outer membrane vesicle formation (Chiang and Tolker-Nielsen, 2010). eDNA is known to accumulate in many Gram-negative and Gram-positive bacterial biofilms (Tetz et al., 2009; Chiang and Tolker-Nielsen, 2010).

Extracellular DNA is present in healthy body sites and fluids, such as the gastrointestinal tract, blood, milk, secretions, and likely on mucosal surfaces (Vlassov et al., 2007). During infection, eDNA can accumulate due to the heavy recruitment of host immune cells and the production of neutrophil extracellular traps (NETs), as discussed later. Chronic lung infections in persons challenged with cystic fibrosis (CF) are caused by polymicrobial biofilms that are adapted for long-term survival. The sputum from CF patients has very high concentrations of eDNA and is the reason for the use of human recombinant deoxyribonuclease (DNase) as a mucolytic treatment (Shak et al., 1990; Ranasinha et al., 1993). Inhaled DNase (Pulmozyme) has been shown to reduce sputum viscosity, inflammation, and exacerbations, as well as improve lung function and survival (Jones and Wallis, 2010; Konstan and Ratjen, 2012).

### DNA IS A NUTRIENT SOURCE

Given the abundance of eDNA in the environment, it is not surprising that DNA has a significant influence on bacterial physiology and serves many functions for bacteria. eDNA has been shown to serve as a sole nutrient source of phosphate, nitrogen, and carbon for *Pseudomonas aeruginosa*, *Escherichia coli*, and *Shewanella* spp. (Finkel and Kolter, 2001; Palchevskiy and Finkel, 2006; Pinchuk et al., 2008). We identified a secreted DNase (EddB) that is produced in the presence of low DNA concentrations and under limiting phosphate conditions (Mulcahy et al., 2010). The EddB DNase is required for degradation of eDNA and utilization of DNA fragments or nucleotides as a sole source of carbon, nitrogen, and phosphate (Mulcahy et al., 2010). There is an alkaline phosphatase expressed upstream of the DNase, EddA, which may also be required for phosphorus acquisition from DNA. In *Shewanella oneidensis*, a secreted DNase (ExeM) with significant homology to EddB (34% identity) is also required for utilization of DNA as a nutrient source (Godeke et al., 2011). A number of intracellular ssDNA exonucleases have also been shown to be required for growth using DNA as a sole carbon course (Palchevskiy and Finkel, 2006). DNA uptake also facilitates lateral gene transfer (LGT) and integration of foreign DNA sequences into the genome. Palchevskiy and Finkel (2006) proposed that dsDNA was taken into the cell, similar to the process of DNA uptake for LGT, converted to ssDNA and then degraded by intracellular exonucleases upon entry into the cytoplasm.

### DNA IS A BIOFILM MATRIX POLYMER

Extracellular DNA is required and primarily acts to facilitate attachment, aggregation, stabilization, and maturation of biofilm formation (Chiang and Tolker-Nielsen, 2010). DNase treatment of young *P. aeruginosa* biofilms results in biofilm dissolution, but mature biofilms resist DNase treatment, indicating a role in early biofilm formation (Whitchurch et al., 2002). Accumulation of exopolysaccharide (EPS) in mature biofilms probably accounts for the inability to degrade mature biofilms with exogenous DNase. Mutant strains that accumulated less eDNA during biofilm formation were more destabilized by treatment with sodium dodecyl sulfate (SDS; Allesen-Holm et al., 2006), providing further evidence for a role in biofilm stabilization. Treatment of young biofilms with DNase impaired the development of the cap structures of mushroom-shaped biofilms (Barken et al., 2008). DNase treatment of biofilms formed by Gram-negative or Gram-positive bacteria reduces the biomass, which suggests that eDNA is a ubiquitous DNA polymer (Tetz et al., 2009). The exception to the rule is in *Caulobacter crescentus* where eDNA blocks biofilm formation by binding to the polar holdfast structure, which is required for irreversible attachment (Berne et al., 2010). eDNA has been shown to localize to specific regions of mushroom-shaped microcolonies formed by *P. aeruginosa* in flow-chamber biofilms. In mature microcolonies, eDNA localizes primarily to the stalk structure, at the boundary of the stalk and cap (Allesen-Holm et al., 2006). In unstructured peg-adhered biofilms, eDNA can be visualized throughout thin biofilms with no particular organization (Mulcahy et al., 2008). eDNA has also been shown to be present as a matrix component in biofilms formed *in vivo* during infection with *P. aeruginosa* (Mulcahy et al., 2011; van Gennip et al., 2012), *Haemophilus influenzae* (Jurcisek and Bakaletz, 2007), and *Bordetella* (Conover et al., 2011).

### EXTRACELLULAR DNA TRAPS

Neutrophil extracellular traps were first described in neutrophils, but have since been identified in other immune cell types including eosinophils and mast cells (Brinkmann and Zychlinsky, 2012). NETs can kill Gram-positive and Gram-negative bacteria, fungi, parasites, and viruses (Brinkmann et al., 2004; Urban et al., 2006, 2009; Guimaraes-Costa et al., 2009; Saitoh et al., 2012). Although there are numerous antimicrobial neutrophil components embedded in NETs (Urban et al., 2009), bacterial killing is largely attributed to the antimicrobial activity of histones (Brinkmann et al., 2004). NET killing can be blocked by either dissolving the NET structure with DNase, or by the addition of neutralizing anti-histone antibodies, which block histone antimicrobial activity. The process of NETosis is a novel mechanism of trapping and killing bacteria, as well as limiting bacterial dissemination (Brinkmann and Zychlinsky, 2012; McDonald et al., 2012; Yipp et al., 2012), For the purpose of this review, it is important to note that NET formation during infection is likely a major contribution of DNA accumulation at the site of infection. NET formation has been observed in CF sputum and likely contributes to the accumulation of eDNA during chronic CF lung infections (Marcos et al., 2010; Manzenreiter et al., 2012). Neutrophils are among the first immune cells recruited to the infection site and most of the DNA in the CF lung is derived from neutrophils (Lethem et al., 1990). In plant roots, an eDNA barrier is produced that protects the root from infection and is analogous to eDNA traps of human immune cells (Hawes et al., 2011).

### CATION CHELATION AND ANTIMICROBIAL ACTIVITIES OF DNA

The focus of our initial work was to test the hypothesis that the matrix polymers influence bacterial gene expression. While biofilm polymers are known to have several protective immune evasion functions, we wondered if the matrix polymers also drive unique gene expression profiles that contribute to the phenotypes of cells in biofilms. Our initial observation upon addition of purified DNA exogenously to planktonic cultures was that bacterial growth was inhibited at DNA concentrations greater than 5 mg/ml (Mulcahy et al., 2008). Due to the highly anionic character of DNA, we hypothesized that DNA was a cation chelator and indeed demonstrated that DNA efficiently binds divalent metal cations that including Mg^2+^, Ca^2+^, Mn^2+^, and Zn^2+^ (Mulcahy et al., 2008). In addition, DNA has a rapid antimicrobial killing activity that can be neutralized by pre-incubating DNA with excess cations before exposure to bacteria (Mulcahy et al., 2008). As bacterial surfaces are highly negatively charged and consequently have high levels of Mg^2+^ and Ca^2+^ bound to the surface (Nicas and Hancock, 1980), we suspected that DNA chelated cations from surfaces and disrupted membrane integrity. Using fluorescence microscopy to monitor membrane integrity, we demonstrated that DNA causes major perturbations to the outer and inner bacterial membranes, leading to rapid cell lysis and death. In addition, cells treated with antimicrobial concentrations of DNA released small outer membrane vesicles. This result indicated that DNA can strip sections of outer membrane from the envelope, disrupting outer and inner membrane integrity, resulting in cell lysis. The membrane destabilizing effects of DNA are similar to that of known cation chelator ethylenediaminetetraacetic acid (EDTA). DNA appears to have a broad-spectrum antimicrobial activity against Gram-positive and Gram-negative bacteria (Mulcahy et al., 2008).

### ANTIMICROBIAL PEPTIDE KILLING AND RESISTANCE MECHANISMS

Cationic antimicrobial peptides (APs) are short, amphipathic peptides with broad-spectrum antimicrobial activity produced by the immune systems of most forms of life (Hancock and Sahl, 2006). The mechanism of killing is primarily through membrane binding and disruption, although they also disrupt cytoplasmic processes (Hancock and Sahl, 2006; Kraus and Peschel, 2006). Host defense peptides are another class of short peptides that may not have direct antimicrobial activities, but are protective due to their ability to modulate the innate immune response (Hancock and Sahl, 2006). APs are positively charged and therefore interact with the negatively charged lipopolysaccharide (LPS) in the Gram-negative outer membrane surface. The hydrophobic character permits membrane integration, disruption, and ultimately cell lysis and death. Gram-negative and Gram-positive bacteria alter their membrane charge to resist peptide killing by producing modified phospholipids, LPS, or teichoic acid structures, whose negative charges are masked (Kraus and Peschel, 2006; Anaya-Lopez et al., 2012). Surface modifications that contribute to AP resistance include alanine-modified teichoic acids, highly acylated lipid A, as well as phosphoethanolamine and aminoarabinose-modified lipid A species (Kraus and Peschel, 2006; Moskowitz and Ernst, 2010; Anaya-Lopez et al., 2012). Collectively, these modifications prevent or limit peptide binding or entry and disruption of bacterial membranes. CF isolates of *P. aeruginosa* are known to produce highly acylated lipid A species and aminoarabinose modifications on the 1- and 4′-phosphates of lipid A (Moskowitz and Ernst, 2010).

### DNA-INDUCED EXPRESSION OF THE *pmr* OPERON

The *pmr* genes are required for the covalent addition of aminoarabinose to the 1- and 4′-phosphates of lipid A (Moskowitz and Ernst, 2010), which protects the outer membrane from AP treatment (Johnson et al., 2012), and is required for peptide resistance (Moskowitz et al., 2004; Lewenza et al., 2005). The *pmr* genes are regulated by the PhoPQ and PmrAB systems in *P. aeruginosa*, and in many other Gram-negative organisms including *Salmonella enterica*, *Klebsiella pneumoniae*, and *Yersinia pestis* (Macfarlane et al., 1999; Groisman, 2001; McPhee et al., 2006; Cheng et al., 2010; O’Loughlin et al., 2010). The *P. aeruginosa* PhoQ sensor responds to Mg^2+^ levels and is activated under Mg^2+^ limiting conditions, leading to increased expression of the *pmr* operon. In Mg^2+^-rich conditions, the presence of sub-lethal exposure to APs also induces expression of the *pmr* operon (McPhee et al., 2003), although this adaptive resistance is controlled by the CprRS and ParRS two-component systems (Fernandez et al., 2012).

**FIGURE 1:**
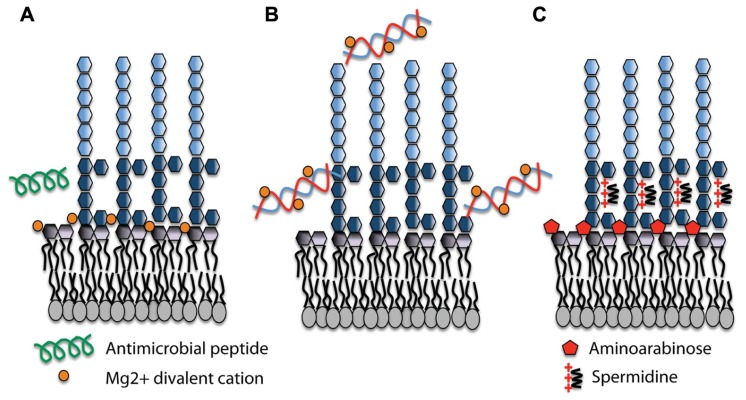
**Lipopolysaccharide (LPS) modifications in the presence of extracellular DNA that contribute to antimicrobial peptide resistance**. **(A)** Divalent metal cations including Mg^2+^ (orange) bind to the negatively charged phosphates of the lipid A moiety of LPS and act to stabilize LPS. Antimicrobial peptides (green) can displace cations and disrupt membrane integrity, leading to cell lysis and death. **(B)** Extracellular DNA binds and sequesters cations from the environment and the membrane. **(C)** In response to limiting Mg^2+^ or cation chelation, the PhoPQ/PmrAB systems are activated leading to the production of covalently attached aminoarabinose to the phosphates of lipid A (red) and the production of polycation spermidine (charge, +3) on the surface, which may bind electrostatically to negative charges in the core oligosaccharide (dark blue) of the O antigen. Both modifications mask the negative charges and protect the outer membrane from peptide damage.

Although DNA prevented growth at higher concentrations, we examined the influence of sub-lethal concentrations of DNA on *pmr* gene expression. In planktonic cultures grown in Mg^2+^ rich media supplemented with DNA, we showed that DNA caused a concentration-dependent induction of the *pmr* operon (*PA3552*–*PA3559*) in *P. aeruginosa* (Mulcahy et al., 2008). DNA induction of this operon can be explained by cation sequestration by DNA, and subsequent activation of the PhoPQ/PmrAB systems. Increased amounts of DNA resulted in more Mg^2+^ sequestered and therefore increasingly higher levels of *pmr* gene expression. **Figure 1** depicts the cation chelating effects of DNA on the structure of LPS in *P. aeruginosa*. Gene induction by DNA can be prevented by the addition of excess cations in combination with DNA, confirming that the cation chelating activity of DNA can be neutralized. We have recently shown that eDNA can also induce expression of the *Salmonella enterica* serovar Typhimurium *pmr* operon and causes increased AP resistance (Submitted), indicating that eDNA may play a general role in activating the PhoPQ system in DNA-rich environments.

### DNA-INDUCED EXPRESSION OF SPERMIDINE SYNTHESIS GENES

A large number of *P. aeruginosa* genes are regulated under Mg^2+^ limiting conditions; some exclusively by PhoPQ and others are controlled by a second Mg^2+^ sensing two-component system PmrAB (McPhee et al., 2006). While the *pmr* operon is directly controlled by both PmrA and PhoP (McPhee et al., 2003, 2006), we identified a three-gene cluster upstream of PmrAB with homology to spermidine synthesis genes *PA4773* (*speD*) and *PA4774* (*speE*) that is controlled exclusively by PmrAB (McPhee et al., 2003). The addition of DNA to planktonic cultures also induced the expression of *PA4773*–*PA4775* in a concentration-dependent manner (Johnson et al., 2012). Mutants in the *PA4773*–*PA4775* genes were sensitive to APs, indicating a potential role in resistance to APs (Lewenza et al., 2005). We confirmed that *PA4773*–*PA4774* were required for spermidine synthesis, which is localized on the bacterial surface (Johnson et al., 2012). Surface and exogenous spermidine protects the outer membrane from APs polymyxin B and CP10A, but also from treatment with other cationic antibiotics including the aminoglycoside gentamicin (Johnson et al., 2012). Polyamines are typically found in the cytoplasm but here we have identified a novel role for polyamines on the bacterial surface. In the presence of eDNA, we proposed that *P. aeruginosa* produces spermidine as an organic polycation replacement for the divalent metal cation Mg^2+^ that functions to mask the negative surface charge and block AP binding (**Figure 1**). Magnesium ions are essential to cross-bridge the core phosphates of lipid A, so it is not surprising that *P. aeruginosa* produces a replacement polycation in the presence of DNA or under Mg^2+^ limiting conditions. Surface polyamines also act as antioxidants and quench reactive oxygen species, thereby protecting the outer membrane from oxidative stress damage to lipids (Johnson et al., 2012).

### DNA-INDUCED ANTIBIOTIC RESISTANCE IN BIOFILMS

To test for a role of DNA-induced expression of the *pmr* genes in biofilm-specific antibiotic resistance, we determined the minimum biofilm eradication concentration (MBEC) in wild type biofilms and in biofilms formed the presence or absence of exogenous DNA (Mulcahy et al., 2008). DNA-enriched biofilms were shown to be eightfold more tolerant to the APs polymyxin B and colistin, and 64- to 128-fold more tolerant to the aminoglycosides gentamicin and tobramycin. Interestingly, planktonic cultures containing exogenous DNA also demonstrated DNA-induced resistance to aminoglycosides and APs (Mulcahy et al., 2008). Exogenous DNA did not have an effect on β-lactam or fluoroquinolones resistance. A mutant in the *pmr* cluster did not exhibit any DNA-induced resistance to APs, indicating that these genes were expressed and required for resistance in DNA-enriched biofilms (Mulcahy et al., 2008). The *pmr* mutant showed an intermediate aminoglycoside resistance phenotype, indicating that the *pmr* aminoarabinose modification also contributed partially to DNA-induced aminoglycoside resistance. It is possible that the anionic eDNA bound positively charged aminoglycosides and provided some protection as a matrix barrier, thus explaining the residual level of resistance in the presence of eDNA. It is known that DNA is capable of binding to aminoglycosides (Ramphal et al., 1988; Purdy Drew et al., 2009) and APs (Bucki et al., 2007). Therefore it is possible that DNA can induce specific resistance mechanisms and also act as a protective matrix absorbing and limiting antimicrobial exposure.

### CONCENTRATION OF eDNA IN BIOFILMS AND INFECTION SITES

An important question that has not been fully answered is to determine if sufficient DNA accumulates in biofilms or during infections, to induce the expression of these protective, AP resistance phenotypes. In microarray studies comparing the gene expression profiles of biofilm to planktonic cultures, the PhoPQ/PmrAB-controlled genes are not among the biofilm-induced genes (Whiteley et al., 2001; Waite et al., 2005). This may be due to an insufficient accumulation of DNA in these particular biofilm model systems, and/or the presence of high Mg^2+^ levels in the growth media used, which can neutralize eDNA and prevent activation of the Mg^2+^ sensing PhoPQ and PmrAB systems. However, a recent paper described a novel regulator of biofilm formation, BfmR, which is required for *P. aeruginosa* to transition to the maturation-1 biofilm developmental stage (Petrova et al., 2011). Biofilms formed by this mutant accumulated more eDNA, which was due to increased bacteriophage-mediated lysis in the *bfmR* mutant. Microarrays were performed on *bfmR* biofilms and both the *pmr* and *PA4774*–*PA4775* genes were induced in *bfmR* biofilms relative to wild type PAO1 (Petrova et al., 2011). This is likely due to the increased eDNA accumulation, but it may be possible that these genes are also controlled by BfmR.

Several papers have reported the *pmr-gfp* gene expression pattern in *P. aeruginosa* flow-chamber biofilms (Haagensen et al., 2007; Pamp et al., 2008). The *pmr* operon is required for colistin resistance in flow-chamber biofilms, but in many of these studies, there was little or no expression of the *pmr* operon in untreated biofilms. This result suggested that there is not sufficient eDNA accumulation in flow-chamber biofilms cultivated under these conditions to influence *pmr* expression. Shortly after colistin treatment, *pmr-gfp* expression was seen in a colistin resistant subpopulation formed on the caps of mushroom-shaped microcolonies (Haagensen et al., 2007). It is known that the presence of APs can induce the *pmr* genes, highlighting an adaptive resistance mechanism whereby the resistance genes are induced by exposure to sub-lethal concentrations of APs (McPhee et al., 2003). The colistin resistant subpopulation is metabolically active, motile, requires various multi-drug efflux pumps, and appears shortly after the early stages of surface attachment (Haagensen et al., 2007; Pamp et al., 2008). Colistin treatment was effective at killing the cells within the inner stalk structures but not the resistant subpopulation on the surface, indicating that colistin penetration is not limited in flow-chamber biofilms, despite the accumulation of eDNA and EPS in these biofilms (Haagensen et al., 2007; Pamp et al., 2008).

Although the total concentration of eDNA can be quantitated in biofilms (Wu and Xi, 2009), the localized concentration may be more important than the overall concentrations. The accumulation of DNA at infection sites is not well documented but sputum from the lungs of persons challenged with CF is known to accumulate DNA at concentrations ranging from <1 to 20 mg/ml (Shak et al., 1990; Ranasinha et al., 1993). There are relatively low Mg^2+^ concentrations in the CF lung (0.08–2 mM; Palmer et al., 2005; Sanders et al., 2006), not high enough to neutralize the cation chelating potential of such high DNA concentrations. Based on the known concentration of DNA and Mg^2+^ in CF lung, it is probable that the PhoPQ/PmrAB-controlled genes are expressed in the CF lung and may contribute to long-term survival in the CF lung. Recently, colistin resistant mutants have been characterized from CF patients and shown to contain gain-of-function PhoQ and PmrB sensor mutations, leading to increased expression of the *pmr* genes (Miller et al., 2011; Moskowitz et al., 2012). This result underscores the importance of these genes in the CF lung, particularly in those patients treated with colistin.

### FUTURE WORK

To date, we have shown that eDNA influences the expression of several genes including a secreted DNase, and at least two operons controlled by the PhoPQ and PmrAB two-component systems. We are currently exploring the global effect of eDNA on bacterial gene expression using a genome-wide transcriptomic method and screening a library of transcription *lux* fusions (Lewenza et al., 2005) to identify novel DNA-induced or repressed genes. While aminoarabinose-modified LPS and surface spermidine both protect the outer membrane and contribute to AP resistance *in vitro*, they may also protect *P. aeruginosa* from APs produced by the innate immune system. It will be important to examine the role of these surface modifications in protecting *P. aeruginosa* from innate immune cells known to produce APs, such as macrophages and neutrophils.

## CONCLUSION

We identified a new property of eDNA as a divalent metal cation chelator, which is required to induce the expression of multiple operons that contribute to decreasing the permeability of the outer membrane to APs and aminoglycosides. *P. aeruginosa* EPS are also anionic polymers with calcium binding properties, indicating that cation binding and sequestration may be a general feature of the biofilm matrix. The anionic charge of DNA may also contribute to antibiotic resistance by binding to cationic antimicrobials and limiting their access to bacterial cells. Since DNA accumulates in the environment, in infection sites and in the biofilm matrix, the influence of DNA on gene expression may contribute to long-term survival in these DNA-rich environments.

## Conflict of Interest Statement

The author declares that the research was conducted in the absence of any commercial or financial relationships that could be construed as a potential conflict of interest.
